# The white blood cell count to mean platelet volume ratio for ischemic stroke patients after intravenous thrombolysis

**DOI:** 10.3389/fimmu.2022.995911

**Published:** 2022-10-03

**Authors:** Yiyun Weng, Yufan Gao, Mingyue Zhao, Tian Zeng, Jiaqi Huang, Haobo Xie, Jiexi Huang, Yiqun Chen, Xiaoya Hu, Jiahan Xu, Jinrong Zhu, Suichai Lin, Tingting Ke, Xiang Li, Xu Zhang

**Affiliations:** ^1^ Department of Neurology, Shandong Provincial Qianfoshan Hospital, Cheeloo College of Medicine, Shandong University, Jinan, China; ^2^ Department of Neurology, The First Affiliated Hospital of Wenzhou Medical University, Wenzhou, China; ^3^ The Second School of Medicine, Wenzhou Medical University, Wenzhou, China; ^4^ The First School of Medicine, School of Information and Engineering, Wenzhou Medical University, Wenzhou, China; ^5^ Department of Emergency, The First Affiliated Hospital of Wenzhou Medical University, Wenzhou, China

**Keywords:** WMR, ischemic stroke, inflammation, intravenous thrombolysis, prognosis

## Abstract

**Background and Purpose:**

White blood cell count to mean platelet volume ratio (WMR) is increasingly recognized as a promising biomarker. However, its predictive capability for acute ischemic stroke (AIS) patients is relatively less researched. The primary aim of this study is to explore its prognostic value in AIS patients after reperfusion regarding 3-month poor functional outcome.

**Methods:**

A total of 549 AIS patients who had undergone vascular reperfusion procedure with complete 3-month follow-up were retrospectively recruited in this study. White blood cell count, mean platelet volume at 24 h of admission were recorded. Stroke severity had been estimated using the National Institutes of Health Stroke Scale (NIHSS) and poor outcome was defined as modified Rankin Scale (mRS) 3–6 at 3 months.

**Results:**

AIS patients with poor functional outcome at 3 months displayed higher WMR. A positive correlation between WMR and NIHSS score was found (r = 0.334, *p* < 0.001). After adjusting potential confounders, WMR was still an independent risk factor for poor prognosis at 3 months (OR = 2.257, 95% CI [1.117-4.564], *p* = 0.023) in multivariate logistic regression model. Subgroup analyses further suggested a significant association between WMR and poor outcome in high baseline NIHSS (per 0.1-point increase: OR = 1.153, 95% CI [1.014-1.312], *p* = 0.030) group. Receiver operating characteristic (ROC) curves analysis was utilized to assess the predictive ability of WMR, indicating a cut-off value of 0.86. A nomogram that includes age, sex, NIHSS on admission, high WMR for predicting 1-year all-cause survival was also developed (C-index = 0.628).

**Conclusions:**

WMR is significantly correlated with stroke severity on admission and is proved to be an important prognostic indicator for AIS outcomes, especially in high NIHSS on admission group. Additionally, the developed nomogram that includes high WMR for predicting 1-year survival provides us with an effective visualization tool.

## Introduction

Acute ischemic stroke (AIS) is a major type of stroke that can cause high mortality and morbidity (1). As a currently accepted and recognized management by rapid vascular reperfusion within 4.5 h of stroke onset, intravenous thrombolysis has been reported to yield a powerful effect on clinical outcomes and reduce disability in AIS patients (2). However, due to the following pathophysiological process after therapy, a proportion of patients are still inclined to develop poor functional outcomes (3). Therefore, seeking an accurate and reliable prognostic indicator to distinguish AIS patients after vascular reperfusion that may have a higher risk of poor functional outcomes is of great clinical value (4).

Systemic inflammation response, in which white blood cell (WBC) plays a vital role, has been proved to be involved in the pathogenesis in ischemic stroke (5). Previous studies among AIS patients have found an association between early elevation of leukocyte and the volume of infarcted area, which has been proven to be positively correlated to poor functional outcomes (6, 7). Also, several studies have suggested that WBC on admission is correlated with greater degree of neurological impairment and unfavorable long-term outcome (8, 9).

Mean platelet volume (MPV), which reflects platelet size, can provide information of platelet function and activation (10). Hyperactive platelets play a pivotal role in thrombus formation and propagation (4), which is one of the pathological processes of AIS. In inflammatory conditions, MPV is also associated with increased percentage of large platelets (11). The predictive value of MPV in ischemic stroke has been explored and confirmed in other studies (4, 12).

As a combination of the two above-mentioned biomarkers, white blood cell count to mean platelet volume ratio (WMR) indicates to be more stable and comprehensive regarding the pathology of AIS (13). It has been recognized in other literature as a relatively new marker for prognosis in atherosclerotic diseases and myocardial infarction (14). Moreover, recent evidence suggested an association between platelet-to-white blood cell ratio (PWR), a similar index to WMR, and the National Institutes of Health Stroke Scale (NIHSS) on admission and on 7 days after admission in AIS patients (15). This previous study has also provided important information on the clinical value of the two combined biomarkers. However, the predicting value of WMR in the prognosis of AIS patients still remains less researched. Therefore, in this retrospective observatory cohort study, we sought to analyze whether WMR would be able to indicate patients at high risk for poor outcomes at 3 months after vascular reperfusion.

## Materials and methods

### Study population

In this retrospective study, a total of 549 AIS patients receiving intravenous thrombolysis therapy were consecutively recruited from the First Affiliated Hospital of Wenzhou Medical University from January 2019 to December 2020. The exclusion criteria were set as follows (1): with cancer; (2) with autoimmune diseases; (3) with severe hepatic failure or renal failure; (4) use of steroid or non-steroid anti-inflammatory treatment (14, 16); (5) with infections within 48h after admission.

**Figure 1:**
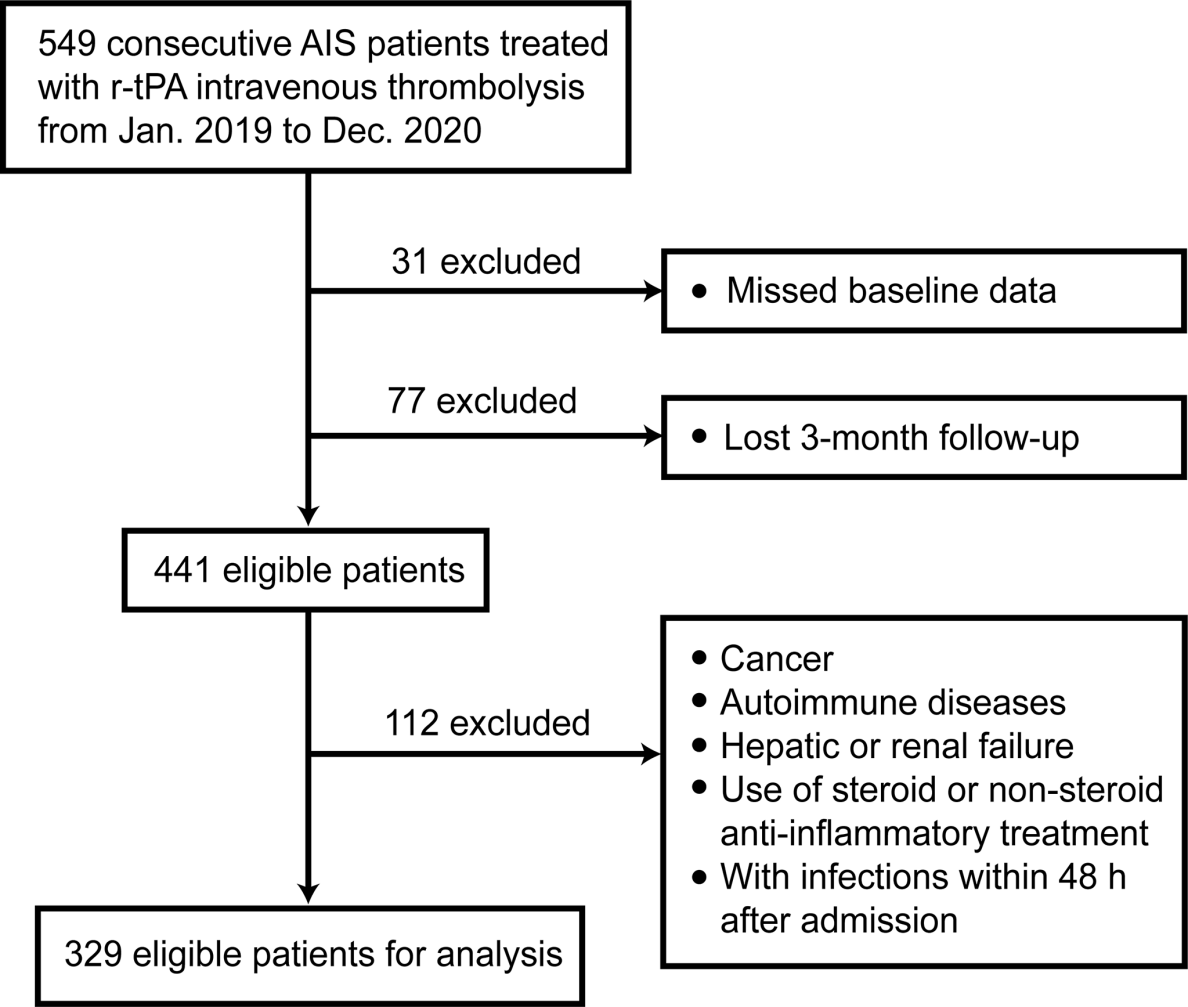
Flow chart for patient selection.

We followed up these patients for 3 months and 1 year after AIS onset. After excluding 108 patients with missing data, there remained 441 patients. Finally, a total of 329 patients were eligible for analysis with complete 3-month follow-up. **Figure 1** shows the exclusion and inclusion procedure in the form of flow chart.

The study was approved by the Ethics Committee of the First Affiliated Hospital of Wenzhou Medical University and was performed in accordance with the Declaration of Helsinki. All patients signed a written informed consent form.

### Data collection

Demographic data (age, sex) and medical history (smoking, alcohol, hypertension, diabetes, atrial fibrillation, coronary artery disease and prior stroke) of patients were obtained from medical records. Serum biomarkers included WBC count, MPV collected at 24 h of admission. WMR was calculated with WBC counts divided by MPV. Stroke severity was measured by NIHSS on admission and 24 h after admission. At 3 months and 1 year after the onset of AIS, the prognoses of patients were assessed using modified Rankin Scale (mRS) through telephone follow-up by two experienced clinicians.

### Clinical assessment

The etiology of AIS was classified based on the Trial of Org 10,172 in Acute Stroke Treatment (TOAST) criteria: large artery atherosclerosis (LAA), small artery occlusion (SAO), cardio-embolism (CE), and others (17). Primary outcome included death or major disability at 3 months, defined as mRS score at 3-6. Secondary outcomes included poor functional outcome at 3 months defined by mRS score 2-6, early neurological improvement (ENI) defined as NIHSS score at 0-1 within 24 h after thrombolysis (18), early neurological deterioration decrease (END) defined as a decrease ≥ 4 points in the NIHSS score (19), death or major disability (mRS 3-6) at 1 year and all-cause mortality at 1 year.

### Statistical analysis

We used SPSS Statistics 26.0.0.0, MedCalc Statistical Software version 18.2.1, GraphPad Prism version 9.0.0 and R version 4.1.3 for plots and statistical analyses. Data were first analyzed for normality of distribution using the Kolmogorov-Smirnov test of normality. Continuous variables with normal distribution were expressed as mean with standard deviation (mean ± SD) while continuous variables with non-normal distribution were expressed as medians and interquartile range (median, IQR). Patients were attributed into high/low WMR groups according to the cut-off value and inter-group differences were compared using Mann-Whitney U test. Participants were then divided into four groups according to WMR quartile (Q1 < 0.581, Q2 0.581-0.728, Q3 0.728-0.919, Q4 > 0.919). Differences of baseline characteristics across WMR quartile were compared using Kruskal-Wallis test. Categorical variables were presented as frequency and percentages (n, %) and differences among groups were compared through chi-squared test or Fisher’s exact test.

Case distribution across quartile-based categories of WMR and mRS score at 3 months was presented using density plots. To test the correlation between WMR and NIHSS, we calculated Spearman’s correlation coefficient. To account for differences in low WMR distributions across stroke subtype, and differences in mRS distribution in high and low WMR groups classified using WMR cutoff value, chi-squared test was performed.

Univariate and multivariate logistic analyses were utilized to demonstrate the association between WMR and primary outcome. The crude model, model 1, was carried out by univariate analysis. In model 2, we adjusted for covariates with a *p*-value < 0.1 in model 1. To further explore the potential predictive ability disparity in different subgroups, stratified analysis was undertaken. With WMR median as a node undergoing binary logistic regression, restricted cubic splines with 3 knots (at 0.6, 0.9 1.2 respectively) were also plotted to demonstrate the correlation between WMR and AIS outcomes.

Receiver operating characteristic (ROC) analysis was employed to determine the optimal cutoff value and the ability of WMR to distinguish poor functional outcomes. Multivariable time-to-event analysis was performed using Cox proportional hazards regression models to develop a nomogram using weighted estimators corresponding to each covariate that was found statistically significant in predicting 1-year mortality using multivariate regression model. A *p-*value of less than 0.05 was considered statistically significant.

## Results

### Characteristics of study patients

A total of 329 AIS patients after intravenous thrombolysis were retrospectively included for analysis in this study, with a median age of 67 years and men accounting for 64.1% (211). The median NIHSS score on admission for all study patients was 6, and median WMR was 0.73. A total of 91 (27.7%) patients had poor functional outcomes at 3 months, 31 patients (9.4%) died during the 1-year follow-up. The clinical characteristics of AIS patients were displayed in **Table 1**. Compared with patents with a good functional outcome, those in the poor outcome group had significantly older age (median age: 72.0 vs. 65.0, *p* < 0.001), higher proportion of hypertension (71.4% vs. 50.4%, *p* = 0.001), higher proportion of atrial fibrillation (AF) (12.1% vs. 5.5%, *p* = 0.039), higher WBC count (median: 9.0 vs. 7.5, *p* < 0.001), higher WMR (median: 0.88 vs. 0.70, *p* < 0.001), higher NIHSS on admission (median: 12.0 vs. 5.0, *p* < 0.001), and at 24 h (median: 13.0 vs. 4.0, *p* < 0.001). Those with a good functional outcome were more likely to be male (71.4% vs. 45.1%, *p* < 0.001) and the small artery occlusion (SAO) subtype (24.8% vs. 4.4%, *p* < 0.001). Furthermore, smoking history (41.2% vs. 26.4%, *p* = 0.013), alcohol drinking history (38.2% vs. 25.3%, *p* = 0.027) were more prevalent in patients with a good outcome in comparison with those with a poor outcome.

**Table 1:** Characteristics of AIS patients with or without poor function outcomes at 90 days.

Characteristics	Total	Functional outcomes		
		mRS 0-2 (n = 238)	mRS 3-6 (n = 91)	*p*
Demographic data				
Age (years)	67.0 (57.0-75.0)	65.0 (55.0-74.0)	72.0 (62.0-80.0)	< 0.001
Sex, (male, n%)	211 (64.1)	170 (71.4)	41 (45.1)	< 0.001
Stroke risk factors, n (%)				
Smoking	122 (37.1)	98 (41.2)	24 (26.4)	0.013
Alcohol	114 (34.7)	91 (38.2)	23 (25.3)	0.027
Hypertension	185 (56.2)	120 (50.4)	65 (71.4)	0.001
Diabetes	55 (16.7)	34 (14.3)	21 (23.1)	0.056
AF	24 (7.3)	13 (5.5)	11 (12.1)	0.039
CAD	13 (4.0)	10 (4.2)	3 (3.3)	1.000
Prior stroke	31 (9.4)	19 (8.0)	12 (13.2)	0.148
Serum biomarkers				
WBC count, (×10^9^/L)	7.9 (6.5-10.0)	7.5 (6.1-9.6)	9.0 (7.5-11.2)	< 0.001
MPV, (fL)	10.9 (10.2-11.5)	10.9 (10.2-11.4)	11.0 (10.1-11.8)	0.924
WMR	0.73 (0.58-0.92)	0.70 (0.56-0.86)	0.88 (0.66-1.07)	< 0.001
Clinical features				
NIHSS on admission	6.0 (3.5-11.0)	5.0 (3.0-7.0)	12.0 (9.0-17.0)	< 0.001
NIHSS at 24h	5.0 (3.0-11.0)	4.0 (2.0-6.0)	13.0 (9.0-20.0)	< 0.001
DNT	43.5 (33.2-55.1)	42.5 (32.7-52.9)	46.1 (34.9-59.6)	0.094
Stroke subtype, n				< 0.001
LAA, n (%)	136 (41.3)	93 (39.1)	43 (47.3)	
SAO, n (%)	63 (19.1)	59 (24.8)	4 (4.4)	
CE, n (%)	83 (25.2)	53 (22.3)	30 (33.0)	
Others, n (%)	47 (14.3)	33 (13.9)	14 (15.4)	
Reperfusion therapy				< 0.001
IVT, n (%)	277 (84.2)	211 (88.7)	66 (72.5)	
Bridging therapy, n (%)	52 (15.8)	27 (11.3)	25 (27.5)	

mRS, modified ranking scale; WMR, white blood cell to mean platelet volume ratio; AF, atrial fibrillation; CAD, coronary artery disease; WBC, white blood cell; MPV, mean platelet volume; NIHSS, national institute of health stroke scale; LAA, large artery atherosclerosis; SAO, small artery occlusion; CE, cardio-embolism; IVT, intravenous thrombolysis.

As illustrated in **Table 2**, it was found that smoking, WBC count, MPV, NIHSS on admission and at 24 h, SAO subtype distribution, mRS score 3-6, 2-6, mRS 3-6 at 1 year mortality rate at 1 year differed by WMR quartile.

**Table 2:** Comparisons of baseline characteristics and outcomes between groups divided according to WMR quartile.

	WMR				
Characteristics	Q1 (< 0.581)	Q2 (0.581-0.728)	Q3 (0.728-0.919)	Q4 (> 0.919)	*p*
n	82	84	81	82	
Demographic data					
Age, (years)	68.0 (58.0-75.0)	68.0 (58.8-77.8)	67.0 (555-73.5)	63.5 (56.0-75.0)	0.367
Sex, (male, n%)	55 (67.0)	48 (57.1)	56 (69.1)	52 (63.4)	0.393
Stroke risk factors, n (%)					
Smoking	29 (35.3)	24 (28.5)	41 (50.6)	28 (34.1)	0.025
Alcohol	33 (40.2)	23 (33.3)	29 (35.8)	24 (29.2)	0.514
Hypertension	41 (50.0)	51 (60.7)	48 (59.2)	45 (54.8)	0.504
Diabetes	12 (14.6)	13 (15.4)	16 (19.7)	14 (17.0)	0.827
AF	7 (8.5)	8 (9.5)	1 (1.2)	8 (9.7)	0.066
CAD	1 (1.2)	4 (4.7)	5 (6.1)	3 (3.6)	0.413
Prior stroke	11 (13.4)	8 (9.5)	9 (11.1)	3 (3.6)	0.138
Serum biomarkers					
WBC count, (×10^9^/L)	5.5 (4.6-6.2)	7.2 (6.7-7.8)	8.9 (8.2-9.7)	11.5 (10.6-13.5)	< 0.001
MPV, (fL)	11.2 (10.7-11.9)	11.1 (10.3-11.6)	10.6 (10.1-11.4)	10.5 (10.0-11.2)	< 0.001
Clinical features					
NIHSS on admission	4.0 (3.0-5.0)	6.0 (4.0-10.0)	6.0 (4.0-10.0)	10.0 (5.0-16.0)	< 0.001
NIHSS at 24h	4.0 (2.0-5.0)	6.0 (3.0-10.0)	5.0 (3.0-9.8)	9.0 (4.0-15.0)	< 0.001
DNT	44.3 (33.5-56.6)	41.1 (30.9-54.3)	47.6 (34.9-57.6)	42.8 (33.4-51.5)	0.502
Stroke subtype, n					
LAA, n (%)	29 (35.3)	30 (35.7)	39 (48.1)	38 (46.3)	0.195
SAO, n (%)	21 (25.6)	19 (22.6)	16 (19.7)	7 (8.5)	0.031
CE, n (%)	19 (23.1)	24 (28.5)	15 (18.5)	25 (30.4)	0.282
Others, n (%)	13 (15.8)	11 (13.0)	11 (13.5)	12 (14.6)	0.959
Reperfusion therapy					
IVT, n (%)	77 (93.9)	71 (84.5)	73 (90.1)	56 (68.2)	< 0.001
Bridging therapy, n (%)	5 (6.0)	13 (15.4)	8 (9.8)	26 (31.7)	< 0.001
Outcome					
mRS 3-6 at 90 days, n (%)	10 (12.1)	22 (26.1)	22 (27.1)	37 (45.1)	< 0.001
mRS 2-6 at 90 days, n (%)	19 (23.1)	33 (39.2)	29 (35.8)	48 (58.5)	< 0.001
ENI	12 (14.6)	13 (15.4)	7 (8.6)	7 (8.5)	0.375
END	3 (3.6)	7 (8.3)	7 (8.6)	10 (12.1)	0.221
mRS 3-6 at 1 year, n (%)	3 (3.6)	14 (16.6)	15 (18.5)	25 (30.4)	< 0.001
Mortality at 1 year, n (%)	0 (0)	5 (5.9)	10 (12.3)	16 (19.5)	< 0.001

WMR, white blood cell to mean platelet volume ratio; AF, atrial fibrillation; CAD, coronary artery disease; WBC, white blood cell; MPV, mean platelet volume; NIHSS, national institute of health stroke scale; DNT, door to needle time; LAA, large artery atherosclerosis; SAO, small artery occlusion; CE, cardio-embolism; IVT, intravenous thrombolysis; mRS, modified ranking scale; ENI, early neurological improvement; END, early neurological deterioration.

### The correlation between WMR and clinical status

**Figure 2:**
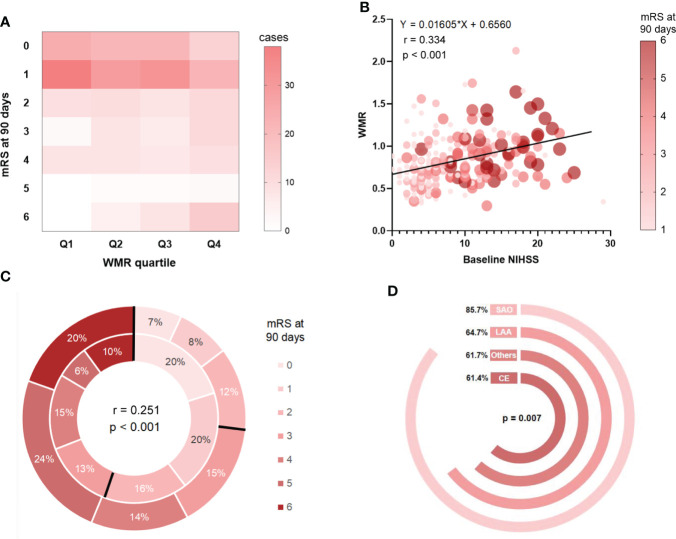
Correlation analyses between WMR and patient features and outcome. **(A)**: Case distribution across quartile-based categories of WMR and mRS score at 90 days. **(B)**: The relationships between WMR and baseline NIHSS as well as mRS at 3 months. **(C)** mRS distribution at 3 months for high WMR group v.s. low WMR group. The outer layer represented distribution of mRS score in high WMR group while the inner layer represented that in low WMR group. **(D)**: Ratio of low WMR group in different stroke subtypes.

We plotted a density plot to display patient distribution cross WMR quartile. The intensity of color represented the frequency of cases. It can be observed in **Figure 2A**, that in general, 200 out of the total 329 cases had the outcome mRS 0-1 (60.8%). In Q1, cases with mRS score at 1 took up the highest proportion (46.3%), while in Q4, 15 cases developed a mRS score at 6 (18.3%). The case distribution across WMR quartile was statistically significant (*p* < 0.001). Correlation analysis demonstrated a significant linear trend between WMR and baseline NIHSS (r = 0.334, *p* < 0.001). The size of circles represented the relative weighting of mRS score at 3 months. It can be seen in **Figure 2B**, that patients with higher WMR showed a higher NIHSS score on admission and a higher mRS score at 3 months. We further investigated the difference in distribution of mRS score between patients in high WMR group (the outer layer of the circle) and low WMR group (the inner layer). High WMR was found to be positively related to death or major disability (mRS 3-6) (r = 0.251, *p* < 0.001), and proportion of mRS 3-6 in high WMR group was more prominent compared with that in low WMR group (**Figure 2C**). In addition, we plotted **Figure 2D**, to visually display the ratio of low WMR group in different stroke subtypes. Chi-squared test verified a significant difference in low WMR distributions across stroke subtype (*p* = 0.007), and proportion of low WMR was reported to be the largest in SAO subtype (85.7%).

### Predictive value of WMR for primary outcome

**Figure 3:**
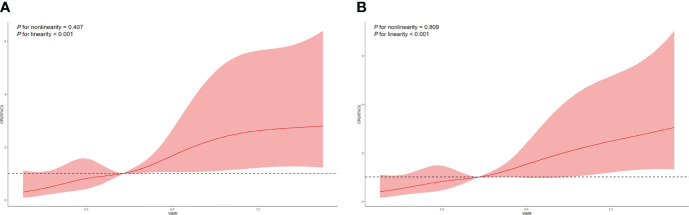
Restricted cubic spline regression analyses of WMR and poor outcomes. **(A)** mRS 3-6 at 3 months. **(B)** mRS 2-6 at 3 months.

As shown in model 1 conducted under the univariate regression analyses, age, smoking, alcohol, hypertension, AF, NIHSS on admission, stroke subtypes and high WMR (OR = 3.169, 95% CI [1.913-5.249], *p* < 0.001) were found significantly associated with primary outcome (mRS 3-6) at 3 months. To determine whether high WMR possessed the ability to independently indicate the prognosis for poor outcome at 3 months, multivariate logistic regression was then performed. In model 2, after adjusting for potential confounders with a *p*-value < 0.1 in model 1, high WMR remained to have an independent association with poor 3-month function outcome (OR = 2.257, 95% CI [1.117-4.564], *p* = 0.023) (**Table 3**). Univariate logistic regression model with restricted cubic splines revealed a positive association between WMR level and the odds of 3-month poor outcomes, including mRS 3-6 and mRS 2-6 (both *p* for linearity < 0.001, **Figure 3**).

**Table 3:** Univariate and multivariate logistic regression analysis for 3-month poor outcome.

	Model 1		Model 2	
Variables	OR (95% CI)	*p*	OR (95% CI)	*p*
Age (years)	1.050 (1.027-1.073)	< 0.001	1.033 (1.002-1.064)	0.034
Sex (male, n%)	0.328 (0.199-0.541)	< 0.001	0.283 (0.121-0.661)	0.004
Vascular risk factors, n (%)				
Smoking	1.954 (1.147-3.330)	0.014	0.986 (0.385-2.526)	0.976
Alcohol	1.830 (1.066-3.141)	0.028	1.386 (0.547-3.508)	0.491
Hypertension	2.458 (1.460-4.139)	0.001	1.290 (0.628-2.652)	0.488
Diabetes	1.800 (0.980-3.306)	0.058	1.306 (0.538-3.167)	0.555
AF	2.380 (1.025-5.526)	0.044	1.203 (0.247-5.865)	0.819
CAD	0.777 (0.209-2.891)	0.707		
Prior stroke	1.751 (0.813-3.771)	0.152		
NIHSS on admission	1.267 (1.197-1.342)	< 0.001	1.213 (1.136-1.296)	< 0.001
DNT	1.012 (0.999-1.024)	0.068	1.014 (0.997-1.031)	0.102
Stroke subtype, n (%)				
LAA	1.000	0.002	1.000	0.049
SAO	0.147 (0.050-0.430)	< 0.001	0.131 (0.026-0.654)	0.013
CE	1.224 (0.689-2.177)	0.491	0.484 (0.197-1.184)	0.112
Others	0.918 (0.226-1.889)	0.815	0.892 (0.342-2.325)	0.815
High WMR	3.169 (1.913-5.249)	< 0.001	2.257 (1.117-4.564)	0.023

Model 2: adjusted for age, sex, smoking, alcohol, hypertension, diabetes, atrial fibrillation, NIHSS at admission, DNT, stroke subtype and high WMR.

OR, odd ratio; CI, confidence interval; AF, atrial fibrillation; CAD, coronary artery disease; WBC, white blood cell; MPV, mean platelet volume; WMR, white blood cell to mean platelet volume ratio; NIHSS, national institute of health stroke scale; DNT, door to needle time; LAA, large artery atherosclerosis; SAO, small artery occlusion; CE, cardio-embolism.

In terms of the ROC analysis for the prediction of poor 3-month outcome using WMR, the cut-off value with the optimal distinguishing capacity of this indicator was 0.86 (a sensitivity of 51.6 and a specificity of 75.2). Additionally, The area under curve (AUC) was 0.662 (95% CI [0.608-0.713], *p* < 0.001) (**Figure S1**).

### Subgroup analyses

In subgroup analyses stratified by age, sex, smoking, alcohol, hypertension, diabetes, NIHSS on admission and stroke subtype (LAA), an attenuated association between WMR and poor functional outcomes in patients with lower NIHSS score on admission was identified (**Table 4**). WMR per 0.1-point increase was independently associated with poor functional outcomes in age ≥ 67 (OR = 1.207, 95% CI [1.015–1.436], *p* = 0.034) and NIHSS on admission ≥ 6 (OR = 1.153, 95% CI [1.014–1.312], *p* = 0.030) groups (**Table 4**).

**Table 4:** Subgroup analyses of the association between WMR and primary outcome (death or major disability).

Subgroups	Primary outcome: death or major disability (mRS 3-6)				
	N	Events (%)	WMR* OR (95% CI)	*p*
Age (≥ 67)				
Yes	173	62 (35.8)	1.207 (1.015-1.436)	0.034
No	156	29 (18.6)	0.975 (0.818-1.161)	0.773
Interaction				0.568
Sex (Male)				
Yes	211	41 (19.4)	1.059 (0.894-1.253)	0.509
No	118	50 (42.4)	1.198 (0.993-1.445)	0.059
Interaction				0.217
Smoking				
Yes	122	24 (19.7)	1.088 (0.879-1.348)	0.439
No	207	67 (32.4)	1.148 (0.980-1.346)	0.088
Interaction				0.262
Alcohol				
Yes	114	23 (20.2)	1.109 (0.918-1.339)	0.285
No	215	68 (31.6)	1.133 (0.975-1.317)	0.104
Interaction				0.554
Hypertension				
Yes	185	65 (35.1)	1.064 (0.905-1.251)	0.454
No	144	26 (18.1)	1.140 (0.885-1.467)	0.310
Interaction				0.340
Diabetes				
Yes	55	21 (38.2)	1.164 (0.671-2.020)	0.589
No	274	70 (25.5)	1.128 (0.998-1.276)	0.054
Interaction				0.654
NIHSS on admission (≥ 6)				
Yes	169	79 (46.7)	1.153 (1.014-1.312)	0.030
No	160	12 (7.5)	1.031 (0.788-1.348)	0.825
Interaction				0.036
Stroke subtype (LAA)				
Yes	136	43 (31.6)	1.079 (0.912-1.277)	0.373
No	193	48 (24.9)	1.125 (0.956-1.324)	0.158
interaction				0.639

The above model adjusted for age, sex, smoking, alcohol, hypertension, diabetes, atrial fibrillation, NIHSS at admission and stroke subtype. In each case, the model is not adjusted for the stratification variable. WMR*, per 0.1 point increase. OR, odd ratio; CI, confidence interval; mRS, modified ranking scale; AF, atrial fibrillation; NIHSS, national institute of health stroke scale; SAO, small artery occlusion; CE, cardio-embolism; OR, odd ratio; CI, confidence interval.

### Predictive value of WMR for all-cause mortality

**Figure 4:**
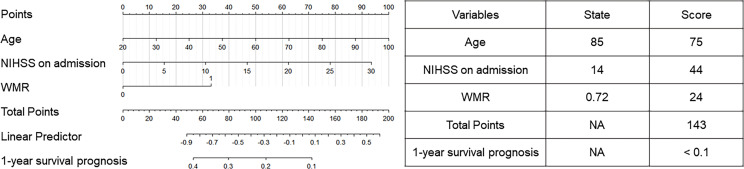
Instructions for using nomogram.

Univariate and multivariate analyses (**Table S2**) were performed to spot prognostic factors to build the nomogram. In the univariate analysis, age (OR = 1.092, 95% CI [1.050–1.135], *p* < 0.001), sex (OR = 0.383, 95% CI [0.176–0.836], *p* = 0.016), hypertension (OR = 3.843, 95% CI [1.502–9.833], *p* = 0.005), AF (OR = 8.064, 95% CI [2.290–28.400], *p* = 0.001), NIHSS on admission (OR = 1.260, 95% CI [1.163–1.364], *p* < 0.001) and high WMR (OR = 4.007, 95% CI [1.795–8.943], *p* = 0.001) were found to be independent risk factors for 1-year mortality. After including these factors with *p* < 0.05 into the multivariate logistic regression model, age (OR = 1.076, 95% CI [1.028–1.126], *p* = 0.002), NIHSS on admission (OR = 1.190, 95% CI [1.085–1.305], *p* < 0.001), and high WMR (OR = 3.251, 95% CI [1.145–9.227], *p* = 0.027) still remained significant. By including those, a nomogram was constructed (**Figure 4**) and was evaluated to possess a relatively high accuracy (C-index = 0.628). Table on the right showed the use of the nomogram with one death case as an example. For an 85-year-old patient with NIHSS score on admission at 14 and WMR at 0.72, the total score (143) was calculated with corresponding scores on the nomogram and presented the 1-year survival prognosis score (< 0.1).

## Discussion

This study set out to explore the association between the relatively new biomarker WMR and the functional outcomes in patients with AIS after intravenous thrombolysis therapy. Overall, our current study mainly established that (1) WMR was significantly higher in patients with poor functional outcomes; (2) There was a linear relationship between WMR and NIHSS on admission, mRS 3-6, and mRS 2-6; (3) After adjusting for potential confounders, high WMR was still significantly correlated with primary outcome; (4) WMR was independently associated with poor functional outcome in older age and high NIHSS score on admission group, and the association was attenuated in patients with lower NIHSS score on admission. (5) WMR could be utilized to predict 1-year survival with a nomogram.

Vascular reperfusion as a therapy to restore the blood supply and limit the brain damage plays an important role in the management of AIS. Currently, available types of therapy to realize the purpose of vascular reperfusion in AIS patients mainly include intravenous thrombolysis and endovascular treatment (20). However, due to the complex stroke pathobiology, these strategies may have limited effectiveness and induce serious side effects (21). In the past few decades, blood-based biomarkers for prognosis of poor functional outcomes following reperfusion therapy in AIS patients has been an object of research (22). Therefore, it is of great importance to identify reliable and accurate biomarkers that could facilitate disease management and clinical decision making.

The impact of inflammatory processes in the progression of AIS has been widely recognized (5). Previous studies have validated that an elevated WBC count is independently associated with stroke severity on admission, poor functional outcomes at discharge, and mortality rates in AIS patients (6). Our data suggested a significantly elevated WBC count at 24 h of admission in the group with poor functional outcome, which also supported the prognostic capability of WBC count.

Platelets play a role in the formation of atherosclerosis and atherothrombosis, which is a critical process during the pathophysiology of stroke (23). They not only participate in clot formation, but are also involved in inflammatory processes (24). During the inflammatory course, the proportion of larger platelets was found to increase, probably because of a synthesis of factors that promote coagulation and inflammation, and a release of platelet stored in the spleen (11). At the same time, these platelets are fast recruited to the inflammation site where they might be activated and worn down, which can possibly account for the decrease of MPV in patients in an inflammation condition (25). Besides, ischemia-reperfusion injury (IRI) is also a prominent pathological process after reperfusion therapy in AIS patients, which may result in a consumption and sequestration of platelets, and thus be a part of the explanation (24).

WMR as a composite marker comprised of WBC count and MPV, indicates a promising value for the prognosis of cardiovascular outcomes (14). The interaction between the two single biomarkers is suggested to be related to the inflammatory response and stroke infarction development, where their counts are mutually affected (15). The elevation of WMR has been demonstrated by other study to reliably identify lower extremity artery disease (LEAD) patients at high risk for chronic limb-threatening ischemia (CLTI), and an association with the endpoint of stroke, which has not been elaborated (13). Additionally, WMR has also been researched in the prediction for patients with non-ST elevation myocardial infarction (14). Though previous research has identified an association between PWR with the severity of AIS and also divided patients according to stroke etiology, it did not examine the long-term prognostic value of the index and did not consider reperfusion therapy (15). In the current study, we explored the predictive value of WMR for different outcomes in a group of patients that all had undergone reperfusion therapy and conducted a further subgroup analysis. However, the prognostic value of WMR in AIS remains less researched. Therefore, more trials concentrating on AIS patients regarding the efficacy of WMR are supposed to be carried out in the future to validate our findings.

Our findings of the elevation in WMR might be explained by an increase in the WBC count and an decrease in the MPV in the inflammation process, which can provide indications for the disease development for AIS patients (13). Sensitivity and specificity analysis demonstrated that compared to WBC as an independent predictor, the value of sensitivity decreased but specificity for prognosis improved after adding MPV to form the WMR parameter, which reduces the probability of false positive rate (**Table S1**). At the same time, it can be easily calculated using the two cheap and available biomarkers from a complete blood cell count test. Thus, it can be of help for clinicians to make relatively accurate prognosis for AIS patients at an early stage.

However, it should be noted that several limitations still exist in our study. First, considering the retrospective and observational nature of this study, it was unable to determine the causality between WMR and poor functional outcomes. Second, the sample size was relatively small. Among the 549 patients who were initially enrolled under the inclusion criteria, 31 (5.6%) patients were excluded for missing baseline data and 77 (14.0) were excluded for the loss of 3 months follow-up. Partly due to the sample size, the ROC curve for the prediction of a 3-month poor outcome using WMR did not reach a high prediction capability (AUC 0.662, sensitivity 51.6, specificity 75.2), which needs to be further researched with a larger data base. Additionally, this study was monocentric so selection bias might still exist and an external validation of the nomogram could not be conducted.

## Conclusion

Our study demonstrated that there is a linear correlation between WMR and NIHSS on admission. High WMR is positively related to a poor functional outcome, and possesses a prognostic potential for AIS outcomes. The predictive capability is especially prominent in older and neurologically more severe groups. Furthermore, as an easily obtainable and cost-effective parameter, WMR can also be utilized to predict 1-year mortality.

## Data availability statement

The raw data supporting the conclusions of this article will be made available by the authors, without undue reservation.

## Ethics statement

The studies involving human participants were reviewed and approved by the First Affiliated Hospital of Wenzhou Medical University. The patients/participants provided their written informed consent to participate in this study.

## Author contributions

XL and XZ conceptualized this work. XL and XZ supervised the study. YW, YG, MZ, TZ, JH, HX, JH, YC, XH, JX, JZ, SL, TK acquisition of data, YW, YG, MZ performed the statistical analysis and interpreted data. YW, YG, MZ prepared the manuscript. XL, XZ, YW, YG, MZ, TZ, JH, HX, JH, YC, XH, JX, JZ, SL, TK revised the manuscript. All authors approved the protocol.

## Acknowledgments

We sincerely thank the participating hospitals, patients, their families and colleagues who have provided valuable suggestions for this study.

## Conflict of interest

The authors declare that the research was conducted in the absence of any commercial or financial relationships that could be construed as a potential conflict of interest.

## Publisher’s note

All claims expressed in this article are solely those of the authors and do not necessarily represent those of their affiliated organizations, or those of the publisher, the editors and the reviewers. Any product that may be evaluated in this article, or claim that may be made by its manufacturer, is not guaranteed or endorsed by the publisher.
