# Supplementary material for: The white blood cell count to mean platelet volume ratio for ischemic stroke patients after intravenous thrombolysis
Source: Front Immunol. 2022 Oct 3;13:995911. doi: 10.3389/fimmu.2022.995911 (PMC9574706; doi:10.3389/fimmu.2022.995911)
Supplement: Supplementary file 1 [file DataSheet_1.pdf]

SUPPLEMENTARY MATERIALS

The White Blood Cell Count to Mean Platelet Volume Ratio: A Now Promising Biomarker for Ischemic Stroke Patients after Vascular Reperfusion

Short Title: WMR in AIS

Authors

Yiyun Weng[1,2§], Yufan Gao[2,3§], Mingyue Zhao[2§], Tian Zeng[2,4], Jiaqi Huang[2,4], Haobo Xie[2,4], Jiexi Huang[2,4], Yiqun Chen[2,4], Xiaoya Hu[2,3], Jiahan Xu[2,4], Jinrong Zhu[2,3], Suichai Lin[5], Tingting Ke[5], Xiang Li[2], Xu Zhang[2]

Institutions:

[1]Department of Neurology, Shandong Provincial Qianfoshan Hospital, Cheeloo College of Medicine, Shandong University, Jinan, China.

[2]Department of Neurology, The First Affiliated Hospital of Wenzhou Medical University, Wenzhou, China.

[3]The Second School of Medicine, Wenzhou Medical University, Wenzhou, China.

[4]The First School of Medicine, School of Information and Engineering, Wenzhou Medical University, Wenzhou, China.

[5]Department of Emergency, The First Affiliated Hospital of Wenzhou Medical University, Wenzhou, China.

§Co-first author: Yiyun Weng, Yufan Gao and Mingyue Zhao

Corresponding author: Xu Zhang and Xiang Li

Xu Zhang, Department of Neurology, The First Affiliated Hospital of Wenzhou Medical University, Nanbaixiang Street, Ouhai District, Wenzhou 325000, Zhejiang, China. E-mail: drzhangxu@126.com. Tel: +86-0577-55579371. Fax: +86-0577-55579318.

Xiang Li, Department of Neurology, The First Affiliated Hospital of Wenzhou Medical University, Nanbaixiang Street, Ouhai District, Wenzhou 325000, Zhejiang, China. E-mail: wzmulixiang@126.com.

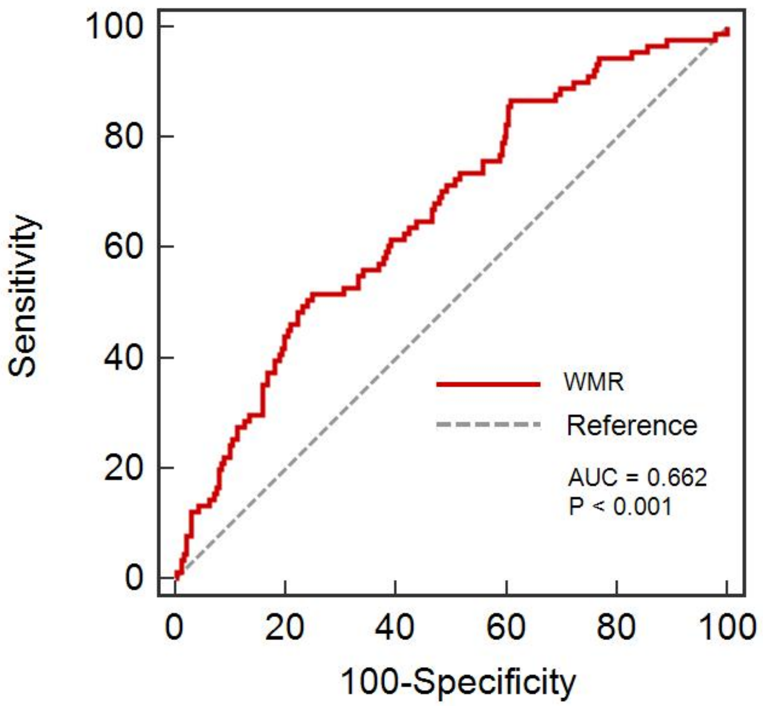

Figure S1. Receiver operator characteristic curves for the prediction of 3-month poor outcome using WMR.

Abbreviations: WMR, white blood cell to mean platelet volume ratio, AUC: area under curve.

Table S1. Sensitivity and specificity analysis for WBC, MPV and WMR respectively.

	Sensitivity	Specificity
WBC	78.0	50.0
MPV	27.5	83.6
WMR	51.6	75.2

Abbreviations: WBC, white blood cell; MPV, mean platelet volume; WMR, white blood cell to mean platelet volume ratio.

Table S2. Univariate and multivariate logistic regression analysis for 1-year mortality.

	Univariate Analysis		Multivariate Analysis	
Variables	OR (95% CI)	*p*	OR (95% CI)	*p*
Age, (years)	1.092 (1.050-1.135)	< 0.001	1.076 (1.028-1.126)	0.002
Sex (male, n%)	0.383 (0.176-0.836)	0.016	0.667 (0.242-1.840)	0.434
Vascular risk factors, n (%)				
Smoking	0.874 (0.387-1.975)	0.747		
Alcohol	0.594 (1.502-9.833)	0.256		
Hypertension	3.843 (1.502-9.833)	0.005	2.443 (0.762-7.829)	0.133
Diabetes	1.801 (0.732-4.433)	0.200		
AF	8.064 (2.290-28.400)	0.001	4.713 (0.771-28.818)	0.093
CAD	3.908 (0.626-24.411)	0.145		
Prior stroke	1.887 (0.637-5.593)	0.252		
NIHSS on admission	1.260 (1.163-1.364)	< 0.001	1.190 (1.085-1.305)	< 0.001
DNT	1.006 (0.987-1.025)	0.550		
Stroke subtype, n (%)				
LAA	1.000	0.005		
SAO*				
CE	1.280 (0.133-12.297)	0.001		
Others	2.723 (0.316-23.494)	0.987		
High WMR	4.007 (1.795-8.943)	0.001	3.251 (1.145-9.227)	0.027

Notes: Multivariate Analysis: adjusted for age, sex, hypertension, AF, NIHSS on admission, and high WMR.

Abbreviations: OR, odd ratio; CI, confidence interval; AF, atrial fibrillation; CAD, coronary artery disease; WBC, white blood cell; MPV, mean platelet volume; WMR, white blood cell to mean platelet volume ratio; NIHSS, national institute of health stroke scale; DNT, door to needle time; LAA, large artery atherosclerosis; SAO*, small artery occlusion, patients in this category did not develop the outcome of death at 1 year; CE, cardio-embolism.
